# Breaking Free of Control: How Conventional T Cells Overcome Regulatory T Cell Suppression

**DOI:** 10.3389/fimmu.2016.00193

**Published:** 2016-05-18

**Authors:** Emily R. Mercadante, Ulrike M. Lorenz

**Affiliations:** ^1^Department of Microbiology Immunology and Cancer Biology, Beirne Carter Center for Immunology Research, University of Virginia, Charlottesville, VA, USA

**Keywords:** conventional T cells, Treg cells, autoimmune disease, immunotherapy, immune tolerance, PI3K/Akt pathway

## Abstract

Conventional T (Tcon) cells are crucial in shaping the immune response, whether it is protection against a pathogen, a cytotoxic attack on tumor cells, or an unwanted response to self-antigens in the context of autoimmunity. In each of these immune settings, regulatory T cells (Tregs) can potentially exert control over the Tcon cell response, resulting in either suppression or activation of the Tcon cells. Under physiological conditions, Tcon cells are able to transiently overcome Treg-imposed restraints to mount a protective response against an infectious threat, achieving clonal expansion, differentiation, and effector function. However, evidence has accumulated in recent years to suggest that Tcon cell resistance to Treg-mediated suppression centrally contributes to the pathogenesis of autoimmune disease. Tipping the balance too far in the other direction, cancerous tumors utilize Tregs to establish an overly suppressive microenvironment, preventing antitumor Tcon cell responses. Given the wide-ranging clinical importance of the Tcon/Treg interaction, this review aims to provide a better understanding of what determines whether a Tcon cell is susceptible to Treg-mediated suppression and how perturbations to this finely tuned balance play a role in pathological conditions. Here, we focus in detail on the complex array of factors that confer Tcon cells with resistance to Treg suppression, which we have divided into two categories: (1) extracellular factor-mediated signaling and (2) intracellular signaling molecules. Further, we explore the therapeutic implications of manipulating the phosphatidylinositol-3 kinase (PI3K)/Akt signaling pathway, which is proposed to be the convergence point of signaling pathways that mediate Tcon resistance to suppression. Finally, we address important unresolved questions on the timing and location of acquisition of resistance, and the stability of the “Treg-resistant” phenotype.

## Introduction

It is well known that Tregs can employ a diverse repertoire of suppressive mechanisms, including secretion of suppressive cytokines, cytotoxicity, metabolic disruption, and modulation of antigen-presenting cell (APC) function (1). Much work has been devoted to delineating how Treg suppressive mechanisms differ *in vitro* versus *in vivo* (2) and how these mechanisms function within specific tissues to shape immune responses (1, 3). Initially, it appeared that most mouse models of autoimmune diseases featured either qualitative or quantitative abnormalities of the Tregs, rendering them inadequate to suppress autoimmune responses [for more detail, see Ref. (4)]. This conclusion arose from the overwhelming evidence that systemic autoimmunity ensued in the absence of Tregs, as in day 3 thymectomy mouse models (5), *Foxp3* mutation in mice (*scurfy*) (6) and humans (IPEX syndrome) (7), or even in Foxp3 conditional KO mouse models (8, 9). Furthermore, genetic models where key components of Treg function are impaired, such as CTLA-4 KO (10) or IL-10 KO (11) mice, supported the idea that Tregs were necessary for immune tolerance, and were the likely culprits in autoimmune disease. More recently, there have been conflicting reports on whether Treg frequency and/or function is actually reduced in autoimmune disease (12). Despite these discrepancies, both reduced Treg number and/or function remain as possible pathological mechanisms (12, 13). However, compelling evidence acquired over the past decade now suggests that Tcon cells that are refractory to Treg suppression also act as mediators of autoimmune disease in mouse models (14–22) and humans (23–35) (see Table 1). It has been clearly demonstrated that Tcon cells – including naive (also called “Th0”) T cells, differentiated effector T cells, and memory T cells – can become refractory to Treg-mediated suppression both *in vitro* (14–35) and *in vivo* (15–35). Tcon cells can become insensitive to Treg-mediated suppression when the ratio of Tcon cells to Tregs is skewed in favor of Tcon cells, when intracellular signaling pathways have been modified by mutations, or through extracellular signals, such as strong activation or a specific cytokine milieu, that induce Tcon cell-intrinsic changes (4). The latter mechanism refers to potentially pathogenic Tcon cells that have become *resistant* to Treg suppression, a phenomenon, which has been observed in several autoimmune diseases and is the focus of this review.

**Table 1:** **Diseases in which Tcon cells resist Treg-mediated suppression**.

Disease	Subject	Type of effector cell	Suggested mechanism	Study
Juvenile idiopathic arthritis (JIA)	Human	Synovial fluid CD4^+^ CD25^−^	Enhanced activation	Haufe et al. (23)
		Synovial fluid CD4^+^ and CD8^+^ Teff^a^	Akt hyperactivation in response to IL-6/TNFα	Wehrens et al. (24, 25)
Rheumatoid arthritis (RA)	Human	Peripheral blood CD4^+^CD25^−^	Increased TRAIL expression on Teff leading to Treg apoptosis	Xiao et al. (26)
Type 1 diabetes (T1D)	NOD mice	Splenic CD4^+^CD25^−^	ND	You et al. (15)
	DO11.10 RIP-mOVA mice	Lymph node CD4^+^CD25^−^	Increased IL-21	Clough et al. (18)
	NOD mice	Splenic CD4^+^CD25^−^	ND	D’Alise et al. (20)
	NOD mice	Splenic CD4^+^ and CD8^+^ Teff	Reduced ganglioside M1 expression on Teff	Wu et al. (21)
	Human	Peripheral blood CD4^+^CD25^−^	ND	Schneider et al. (27)
		Peripheral blood CD4^+^CD25^−^	ND	Lawson et al. (28)
Systemic lupus erythematosus (SLE)	MRL/lpr and NZB/WF1 mice	Splenic and lymph node CD4^+^CD25^−^	ND	Monk et al. (14)
	MRL/lpr mice	Lymph node CD4^+^CD25^−^	ND	Parietti et al. (19)
	Human	Peripheral blood CD4^+^CD25^−^	ND	Venigalla et al. (29)
		Peripheral blood CD4^+^CD25^−^	ND	Vargas-Rojas et al. (30)
		Peripheral blood CD4^+^CD45RA^−^FoxP3^−^	Akt hyperactivation, upregulation of OX40 and impaired TRAF6 in Teff	Kshirsagar et al. (116)
Experimental autoimmune encephalitis (EAE)	FoxP3.gfp KI mice	CNS CD4^+^GFP^−^	High IL-6 and TNFα	Korn et al. (16)
	C57BL/6 mice	CNS CD4^+^CD25^−^	ND	O’Connor et al. (17)
	B6.SLE mice	Splenic CD4^+^CD25^−^	ND	Wilhelm et al. (22)
Multiple sclerosis (MS)	Human	Peripheral blood CD3^+^ Teff^b^	Accelerated production of IL-6 and higher expression of IL-6R on Teff leads to Akt hyperactivation	Trinschek et al. (31)
		Peripheral blood CD4^+^CD25^−^	Increased IL-6 induction of pSTAT3	Schneider et al. (32)
		Peripheral blood CD4^+^CD25^−^	Increased Granzyme B production by Teff w/TCR activation/IL-6 stimulation, inactivating Tregs	Bhela et al. (33)
Inflammatory bowel disease (IBD)	Human	Lamina propria CD4^+^CD25^−^	Higher expression of Smad7 interfering with TGF-β signaling	Fantini et al. (35)
		Lamina propria CD4^+^CD25^−^	Increased IL-15 in lamina propria	Hmida et al. (34)

*ND, not determined; CNS, central nervous system*.

*^a^Teff – total synovial fluid or peripheral blood mononuclear cells (as indicated) isolated as CD4^+^ or CD8^+^*.

*^b^Teff – contains both CD4^+^ and CD8^+^ Teff cells, isolated as CD3^+^*.

The current body of work on this topic predominantly addresses how Tcon cells escape *in vitro* Treg suppression, and how cells that have already become Treg-resistant *in vivo* can continue to resist suppression *in vitro*. The suppressive mechanisms employed by Tregs *in vitro* appear to be distinct from those used *in vivo* (2), complicating the interpretation of results from *in vitro* or *ex vivo* systems with regard to their applicability *in vivo*. For example, IL-2 is needed for Treg survival and homeostasis *in vivo*, but IL-2 signaling is not only dispensable but also counteracts Treg suppressive function *in vitro* (36). Furthermore, Tregs are anergic and generally non-proliferative *in vitro*, but can expand *in vivo* after antigen encounter (2). Despite these Treg differences, *in vitro* systems have provided insights into the molecular mechanism(s) of Tcon cell resistance to Treg suppression, mechanisms that may also be relevant *in vivo*.

The standard method for measuring Treg suppression of Tcon cells is an *in vitro* suppression assay, wherein suppression is the reduction of Tcon cell proliferation and/or cytokine production compared to Tcon cells in the absence of Tregs. Resistance to suppression, therefore, is defined as an increased proliferation and/or cytokine secretion by Tcon cells in the presence of Tregs compared to that of a control Tcon cell (e.g., from a healthy patient or not treated with a resistance-inducing factor). The use of CFSE or CellTrace proliferation dyes was an important technical advance that allowed investigators to gain more detailed information about Tcon resistance to suppression, which was not initially possible using ^3^H-thymidine incorporation. By labeling Tregs or Tcon cells with separate proliferation dyes, investigators were able to directly measure the proliferation of Tcon cells independent of any Treg proliferation occurring in coculture.

One of the technical difficulties with studies assessing resistance to Treg suppression is that simply modulating exogenous factors in *in vitro* coculture systems simultaneously affects Tregs and Tcon cells, making it difficult to distinguish whether there is impaired Treg function, Tcon cell resistance to suppression, or both. Many murine studies have therefore focused on using genetic models that allow for targeted manipulation of specific molecules or downstream signaling pathways to identify effects on Tcon cells independent of changes to Treg function. For example, in the case of exogenous factors inducing resistance, Tcon cells can be assayed in the presence of Tregs that are genetically modified to be deficient for the respective receptor of that factor (37). These “cross-over” suppression assays can also be applied to human studies in order to assess whether Tcon resistance occurs independent of Treg impairment. In such cases, Tcon cells from patients are compared to healthy control subjects in their ability to resist suppression by healthy Tregs (24). Another method to separate effects of external factors on Tcon versus Treg cells is to pre-treat Tcon or Treg cells alone prior to coculture with a given factor, or with pharmacological inhibitors, and then assess changes in Tcon cell suppression by Tregs. Finally, most studies discussed here have included carefully designed controls to quantify the effects of any given factor on baseline Tcon cell stimulation versus the ability to induce resistance to Treg suppression. Under physiological conditions, the factors that cause Tcon cells to resist suppression often also impact Treg function and/or overall Tcon activation. However, the primary focus of this review is the discussion of factors that have been clearly shown to induce changes in Tcon cells, which allow them to specifically resist suppression.

Early studies laid the foundation for the standard *in vitro* suppression assay by defining the conditions that allowed Tregs to suppress Tcon cells, as well as conditions that allowed Tcon cells to overcome suppression. Provision of strong TCR stimulation *via* platebound anti-CD3 allowed both murine and human Tcon cells to proliferate even in the presence of Tregs, whereas lower concentrations of platebound antibody, or use of soluble anti-CD3 stimulation, allowed Tregs to suppress both proliferation and cytokine production by Tcon cells (38, 39). Additionally, strong costimulatory signals *via* anti-CD28 allowed Tcon cells to resist Treg suppression *in vitro* (38, 40, 41). Physiologically, Tcon cells that only receive signal 1 (TCR stimulation) without concomitant signal 2 (costimulation) will become anergic and/or apoptotic (42). Likewise, for Tcon cells to overcome Treg-imposed restraints and mount a protective response during infection, APCs must upregulate B7 molecules (CD80, CD86) in order to provide Tcon cells with strong costimulatory signals. This paradigm was demonstrated in a murine study by Norment and colleagues, who showed that splenic dendritic cells (DCs), which upon activation express high levels of CD80 and CD86, induced Tcon cells to become refractory to Treg-mediated suppression (43). In contrast, stimulation of Tcon cells by antigen-pulsed B cells or plasmacytoid DCs could only induce Tcon cell proliferation in the absence of Tregs due to lower expression of costimulatory molecules (43). The critical nature of costimulation was confirmed by another study, which found that anti-CD28 increased the number of murine Tcon cells producing IL-2 and accelerated the kinetics of IL-2 production, allowing resistance to Treg suppression (41). Strong antigen dose alone did not alter IL-2 kinetics and did not achieve the same level of Tcon cell resistance to Treg suppression. It was therefore suggested that costimulation allows Tcon cells to resist suppression in a manner distinct from strong TCR signaling alone (41). This is consistent with the concept that costimulatory signals are required for optimal Tcon cell activation during an infectious threat, whereas lack of costimulation may provide a mechanism to maintain peripheral tolerance toward self (44).

These initial *in vitro* studies were the first to demonstrate Tcon resistance to suppression in a situation where Treg suppressive function remained intact. During a pathogenic infection, Tcon cells are provided strong TCR stimulation and costimulation, allowing them to circumvent Treg restraints in order to mount a response. By these rules, a low abundance of self-antigen coupled with weak costimulation favors Treg suppression of self-reactive Tcon cells that escaped negative selection, thereby preventing autoimmune disease. Of course, this ideal balance is not always maintained, and regulatory mechanisms gone awry result in disease.

## Resistance-Inducing Mechanisms

### Extracellular Factors

#### Cytokine Milieu

**Figure 1:**
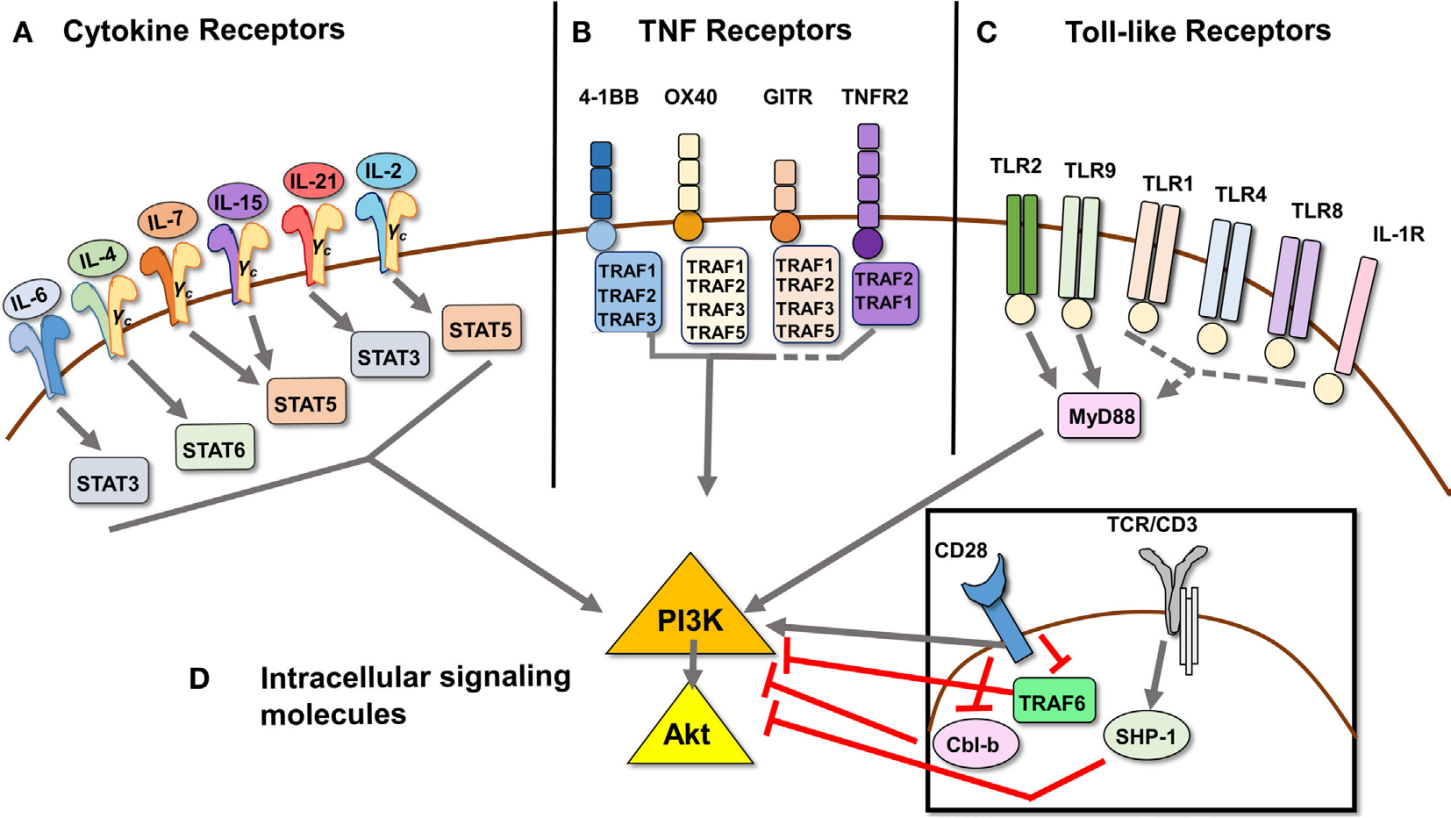
**Signal transduction pathways that mediate Treg resistance converge on the PI3K/Akt pathway**. **(A)** Cytokines IL-6, IL-4, IL-7, IL-15, IL-21, IL-2, and TNFα [ligand for TNFR2, see **(B)** as part of the TNFR superfamily] have been shown to induce Tcon cells to resist Treg suppression. The respective STAT molecule through which each predominantly signals is depicted. **(B)** Signaling through TNF receptors 4-1BB, OX40, GITR, and TNFR2 can induce Tcon cell resistance to Treg suppression, as they provide costimulatory signals similar to CD28 ligation. 4-1BB, OX40, and TNFR2 signaling has been shown to induce PI3K/Akt activation *via* TRAF adaptor proteins, while GITR ligation has not been directly demonstrated to activate the PI3K/Akt pathway. **(C)** Toll-like receptors 1, 2, 4, 8, and 9, as well as IL-1R, also a member of the TLR family, have been shown to induce Treg resistance. Of these, only signaling through TLR2 and TLR9 has been shown to activate the PI3K/Akt pathway *via* recruitment of adaptor protein MyD88, which in turn recruits and activates PI3K *via* its Toll/interleukin-1 receptor domain. **(D)** Intracellular signaling molecules Cbl-b and SHP-1 act as negative regulators downstream of TCR signaling, and genetic deficiency in either induces Treg resistance. Cbl-b enforces the requirement for CD28 costimulatory signaling by inhibiting the recruitment of PI3K to CD28. TRAF6 also negatively regulates activation of PI3K downstream of CD28 costimulation by an as yet undefined mechanism. Dashed lines indicate proposed, but unconfirmed, links between receptors and/or signaling molecules and the PI3K/Akt pathway.

Autoimmune diseases are organ specific or tissue specific and characterized by overproduction of inflammatory cytokines. This is in line with the observation that numerous cytokines associated with autoimmune disease have been found to induce Tcon resistance to Treg suppression in mouse models and human disease: IL-6 (16, 31, 32, 45–49), TNFα (16, 25, 50), IL-15 (51–53), IL-21 (18, 47, 54, 55), IL-1β (56, 57), and IL-4 (58, 59) (Figure 1). Beyond pro-inflammatory cytokines, IL-2 has also long been known to overrule Treg suppression *in vitro* (38, 40, 53).

##### IL-6

Elevated levels of IL-6 have been found to play a pathological role in rheumatoid and juvenile idiopathic arthritis (RA and JIA, respectively), systemic lupus erythematosus (SLE), multiple sclerosis (MS), inflammatory bowel disease (IBD), and allergic asthma (60). Antibody blockade of IL-6 signaling has proven an effective treatment of RA and JIA, and ongoing clinical trials are investigating its use in SLE and Crohn’s disease (60). By far, it has been the most frequently implicated cytokine in inducing Tcon cells to become resistant to Treg-mediated suppression (16, 31, 32, 45–48). Almost all immune cells produce IL-6, and its production is regulated by IL-1, TNFα, interferons, and other stress signals (60). While toll-like receptor (TLR) signaling on monocytes and macrophages leads to IL-6 production during acute inflammation, T cells are the major producers of IL-6 during chronic inflammation (60). Acting in concert with TGF-β, IL-6 induces Th17 cells, thereby preventing the induction of Tregs by TGF-β.

In terms of its role in Tcon resistance, Medzhitov and colleagues demonstrated that activation of murine DCs through TLRs, such as during bacterial infection, could overcome Treg-mediated suppression by producing IL-6. Their results showed that IL-6 alone was necessary but not sufficient to overcome Treg suppression, suggesting that TLR-activated DCs likely produced another cytokine that worked in tandem with IL-6 to induce resistance to Treg suppression (45). It is likely that the DCs also produced TNFα, which has often been found to act along with IL-6 to induce Tcon resistance to Tregs. IL-6 has also been shown to drive Tcon cells to resist Treg-mediated suppression in a chronic inflammatory environment. Tcon cells isolated from the CNS of mice with experimental autoimmune encephalomyelitis (EAE), a model of MS, produced high levels of IL-6 and TNFα and were resistant to Treg suppression (16). IL-6 alone accounted for only half of the observed resistance to suppression, with the other half from an additive effect of TNFα (16). Tcon cells from MS patients, when transferred into NOD/SCID mice, could not be suppressed by healthy donor Tregs *in vivo* (61). Treatment with IFN-β restored susceptibility of MS Tcon cells to Treg suppression, concomitantly lowering IL-6R expression and IL-6 production (61). Like EAE/MS, psoriasis is a Th1-/Th17-driven autoimmune disease characterized by a local inflammatory environment with high levels of IL-6 (46). In addition to Th17 cells producing IL-6 in lesions from psoriasis patients, DCs and endothelial cells produce IL-6 as well, dampening Treg suppression (46).

Mechanistically, IL-6-mediated phosphorylation of STAT3 was found to be crucial in conferring Tcon cells with resistance to Treg suppression. Indeed, high pSTAT3 levels correlated with disease severity in MS (32). Furthermore, IL-6 produced by MS Tcon cells *in vitro* was able to confer “bystander resistance” to Tcon cells from healthy control patients (31). Treatment with pSTAT3 inhibitors restored Tcon cell susceptibility to suppression in cells from MS patients and in healthy control Tcon cells cultured with rhIL-6 (32, 47). On the other hand, IL-27, which also phosphorylates STAT3, could not induce Tcon cell resistance, suggesting signaling downstream of the IL-6-STAT3 axis specifically induces resistance (47). In line with these findings, several studies demonstrated that IL-6-STAT3 signaling led to the activation of Akt (see Figure 1), and that Akt inhibition could restore susceptibility to Treg suppression in Tcon cells from autoimmune disease patients (24, 25, 31). Tcon cells isolated from the synovial fluid (SF) of RA patients have been shown *ex vivo* to resist Treg suppression (26). Although early studies questioned the ability of IL-6 to induce Tcon cell resistance in RA/JIA (51, 53), more recent studies showed that IL-6, in combination with TNFα, allowed Tcon cells to resist Treg suppression. Blockade of both cytokines effectively restored Tcon susceptibility to suppression (24, 25, 62). Thus, the current view is that IL-6, especially in combination with TNFα, is capable of inducing Tcon cells to resist Treg suppression, providing an attractive therapeutic target for reducing inflammation and restoring suppressive balance in autoimmune disease.

##### TNFα

Like IL-6, antibody blockade of TNFα is clinically beneficial for RA and JIA, with it being the first cytokine identified as a therapeutic target in RA (63). TNFα and IL-6 are often produced together in inflammatory settings like the synovium in RA or the CNS in EAE/MS; IL-17, interferons, or other stress factors can drive the production of both cytokines, and TNFα itself can drive the production of IL-6 (16, 60). The complex feedback loops make it harder to dissect the exact role played by a cytokine with regards to Tcon cells acquiring resistance to suppression versus the effects on Tregs themselves. TNFα has been reported to act directly on Tregs to inhibit their suppressive capability (50). When pre-incubating Tregs and Tcon cells with TNFα, Shevach and colleagues observed that TNFα did not affect Tcon cells’ ability to resist suppression but rather inhibited Tregs from subsequently suppressing proliferation and cytokine production of Tcon cells (50). TNFα signaled through TNFRII on Tregs, thereby downregulating the expression of Foxp3 and inhibiting Treg suppressive function (50). An inverse correlation was reported between levels of IL-6 and TNFα in SF from RA patients and the percentage of Foxp3^+^ CD4^+^ Treg cells (62). It is possible that in autoimmune diseases like RA, IL-6 may induce Tcon cells to resist Treg suppression, while TNFα acts on the other side of the equation to further prevent Tregs from suppressing Tcon cells. More recently, however, van Wijk and colleagues demonstrated that TNFα signaling activated Akt in Tcon cells from JIA patients, allowing them to resist Treg suppression, as was seen with IL-6 (24, 25). TNFα blockade directly reduced Tcon cell proliferation, and potentiated suppression by Tregs (25). *In vivo* treatment with a TNFα-blocking antibody did not affect Treg function, but reduced phospho-Akt levels in Tcon cells, thereby reducing their resistance to Treg-mediated suppression (25).

##### Common γ Chain Cytokines: IL-7 and IL-15, IL-2, IL-21, and IL-4

A role for common γ chain (γ_C_) cytokines in Tcon resistance to suppression seems logical, as these cytokines generally promote T cell activation, proliferation, and survival (64). IL-7 and IL-15 have been found at elevated levels in the SF from RA and JIA patients (51, 53), and in the pancreas of murine models of Type 1 diabetes (T1D) (65, 66). Furthermore, IL-7 has been implicated in the pathogenesis of MS and SLE (66). There are several reports of IL-7 and IL-15 inducing human Tcon cell resistance to Treg suppression, either alone (51, 52) or together (53, 54). It appears that both IL-7 and IL-15 act directly on Tcon cells to induce activation of the PI3K/Akt pathway (Figure 1) (52, 67, 68), possibly the mechanism by which Tcon cells become resistant. Thus, IL-7 and IL-15 represent another pair of cytokines that coincide in disease states and can synergize to induce Tcon cells to resist Treg suppression.

Early *in vitro* suppression assays revealed that IL-2 prevented Treg-mediated suppression, though the exact molecular mechanism remains unclear (38, 40). The effects of IL-2 on Tregs *in vitro* and *in vivo* remain complex, and whether IL-2 directly induces Tcon cells to resist Treg suppression is unknown. It is possible that IL-2 signaling induces Treg resistance through activation of the PI3K/Akt pathway (69, 70), but since naive T cells do not express the IL-2 receptor (71), induction of resistance would occur after Tcon cells have become activated. A more recently characterized γ_C_ cytokine, IL-21 has been shown to abrogate Treg suppression of human Tcon cells *in vitro* and *in vivo* (18) without impairing Treg function (54). Importantly, IL-21 did not increase baseline proliferation of Tcon cells, suggesting that resistance to Treg-mediated suppression can occur independently of mechanisms that simply enhance T cell proliferation (47, 54). IL-21 has also been found to promote T cell survival by signaling through the PI3K/Akt pathway (55), likely the mechanism allowing resistance to Treg-mediated suppression. Finally, IL-4 is another common γ_C_ cytokine with the capacity to induce Treg resistance. IL-4 signaling through STAT6 induced murine Tcon cells to resist Treg suppression (58, 59). IL-4 can activate the PI3K/Akt pathway in T cells (72), further suggesting that PI3K/Akt is a potential signaling “hub” for Tcon cell acquisition of Treg resistance.

#### Toll-like Receptors

Toll-like receptors are an essential line of defense against microbial and viral pathogens. Various pathogen-derived ligands signal through TLRs, which recruit adaptor molecules such as MyD88 to trigger the production of pro-inflammatory mediators (73). The goal of TLR signaling is to sense a pathogenic threat and mount innate and adaptive immune responses. TLR ligands can influence T cell responses *via* direct receptor activation or indirectly, by inducing APCs to produce cytokines that affect T cells (74, 75). For example, stimulation of mouse DCs with LPS or CpG (TLR4 and 9 agonists, respectively) induced their production of IL-6, contributing to Tcon cell resistance to Treg suppression (45). Studies of the effects of TLR agonists on mouse and human Treg suppressive function are contradictory [discussed in Ref. (76)], with some suggesting that TLR signaling enhances suppressive function (74, 77), while others show inhibition (37, 78–80), or no change in suppressive function but enhanced Treg survival (76, 81). While it is apparent that TLR signaling directly affects Tregs (75, 82), there is also evidence that TLR signaling can directly induce Tcon cell resistance to suppression.

Both human and murine T cells express mRNA for TLRs 1-9, but protein expression levels vary and depend on the genetic background (in mice) and activation status of the T cell (75, 82, 83). In general, TLR engagement acts as a costimulatory signal to T cells and subsequently activates the PI3K/Akt pathway, consistent with a role in inducing Tcon cells to resist Treg suppression (82, 83). CpG DNA signaling through TLR9 on murine Tcon cells induced IL-2 production, allowing them to escape suppression from MyD88^−/−^ Tregs, which cannot respond to CpG DNA (37, 84). Similarly, TLR2 agonists induced murine Tcon cell resistance to suppression by TLR2^−/−^ Tregs (85, 86), with concurrent activation of the PI3K/Akt pathway (87, 88). Interestingly, human Tcon cells expressing a polymorphism for TLR1 have been shown to resist Treg suppression (89). Like cytokines, TLR signaling impacts both Treg and Tcon cells differentially and therefore must be carefully considered in the context of the overall Treg/Tcon balance. Initially, infection by a bacterial or viral pathogen requires temporary abrogation of Treg suppression in order to allow a T effector response. It has been proposed that early during infection, TLR signals render Tcon cells resistant, and only upon Treg expansion (perhaps due to IL-2 secreted by Tcon cells), the newly increased population is then able to restrict and resolve the inflammatory response (77). Thus, there is likely a complex spatio-temporal regulation of induction of Tcon cell resistance to Treg suppression versus enhancement of Treg suppression by TLR signaling.

#### IL-1β

IL-1β is a potent pro-inflammatory cytokine associated with a wide array of inflammatory states, including some autoimmune diseases (90). Monocytes release IL-1β in response to pathogen or “danger” signals (90). Like TLRs, the IL-1R also contains a Toll/interleukin-1 receptor domain and utilizes MyD88 in signaling (91). Tcon cells and Tregs both express the IL-1R, and IL-1β has been found to enhance the expansion and survival of T cells by activating NFκB and PI3K pathways (91, 92). IL-1β was found to inhibit Treg suppression of murine Tcon cells *in vitro* (56) by acting directly on Tcon cells rather than by impairing Treg function (57). These data suggest that IL-1β may be another factor that, during pathogenic infection, allows Tcon cells to mount a response despite the presence of Tregs. It is possible that IL-1β also induces Tcon cell resistance to suppression in autoimmune disease settings, but this remains to be investigated. IL-1R antibody blockade is being used successfully to treat RA (93), which, in addition to its inflammation-dampening effects, may also reverse Tcon cell resistance to suppression.

#### TNF Receptors

Engagement of certain tumor necrosis factor receptors (TNFRs) on T cells provides costimulatory signals that lead to activation, proliferation, differentiation, and survival (94). In particular, the four TRAF-binding TNFRs described below have been found to render Tcon cells resistant to Treg suppression (95–102). Evidence supports a role, in particular for TRAF2, in activating PI3K/Akt downstream of TNFRs (103), thereby possibly allowing Tcon resistance to Treg suppression. While TNFRs do not contain PI3K-binding motifs, they utilize TRAF adaptor proteins to activate the PI3K pathway (Figure 1). These TNFRs are constitutively expressed on Tregs and become upregulated on activated Tcon cells (100, 104–106). The ligands for these TNFRs are generally expressed on APCs, but can also be induced on other cell types during infection (95, 96, 107). TNFRs, like TLRs, play an important role during an infectious threat by allowing Tcon cells to become efficiently activated in order to mount a response, unrestrained by Tregs. It has therefore been proposed that TNFR ligand expression becomes upregulated during inflammatory conditions and provides costimulatory signals to both Tregs and Tcon cells, with Tcon cells becoming activated, producing IL-2, and resisting Treg suppression. As TNFR ligand levels wane and Tcon cells are no longer able to resist suppression, Tregs can assume control of the immune response (95).

##### GITR

GITR signaling in murine Tcon cells enhanced their proliferation and allowed them to resist Treg-mediated suppression (95). In order to translate this into a therapeutically useful model, Nishikawa and colleagues activated tumor-specific CD4^+^ and CD8^+^ T cells in the presence of GITR signaling, making them become resistant to Treg suppression and able to control tumor growth (108).

##### 4-1BB

Signaling through 4-1BB in murine Tcon cells has been shown to induce proliferation and enhance survival, especially in CD8^+^ T cells (109). Treatment with agonistic 4-1BB antibodies has beneficial effects on CD8^+^ T cell-mediated viral clearance and antitumor immunity (109). *In vitro* studies of 4-1BB signaling have shown a clear role for its CD28-independent costimulation of Tcon cells (109) as well as its ability to induce resistance to Treg-mediated suppression (97–99). Likewise, *in vivo* treatment of mice with anti-4-1BB induced CD8^+^ T cells to become resistant to Treg-mediated suppression in a chronic viral infection model (99). 4-1BB regulation of Treg suppressive function remains controversial (97), but 4-1BBL is capable of *ex vivo* expanding Tregs for therapeutic use (98). Therefore, 4-1BB signaling can induce proliferation of both Tregs and Tcon cells, but directly induces Tcon cells to resist Treg-mediated suppression. Interestingly, 4-1BB signaling has been shown to augment TCR-induced activation of the PI3K/Akt pathway (103, 110), pointing again to a role for PI3K/Akt signaling in Tcon resistance.

##### OX40

OX40 signaling has been reported to both inhibit and enhance Treg suppressive function (100–102, 111–113). In contrast to these conflicting studies, it is clear that OX40 signaling provides costimulation for Tcon cells, promoting their survival and development into memory cells (114). Several studies are in agreement that OX40 signaling in murine Tcon cells induces resistance to Treg-mediated suppression (100–102), possibly *via* PI3K/Akt activation (115, 116). Elevated expression of OX40 is associated with many autoimmune diseases including SLE (116, 117), RA (118), IBD (119–121), and graft-versus-host-disease (GVHD) (122) where Treg resistance has been observed.

##### TNFR2

Originally characterized by its expression on activated/memory Treg cells, TNFR2 marks potently suppressive Tregs present in peripheral lymphoid tissues as well as in tumors, but can also be induced upon TCR activation on Tcon cells (106). While studies have shown that TNF signaling can inhibit Treg suppression, long-term exposure to TNF signaling *via* TNFR2 expanded Tregs and enhanced their suppressive function when given in combination with IL-2 (123). Intriguingly, TNFR2 expression correlated with the suppressive capability of murine tumor-derived Tregs, with TNFR2-negative Tregs being unable to suppress tumor-derived TNFR2-positive Tcon cells (124). This suggested that TNFR2 expression marked a subpopulation of Tcon cells, which were more difficult to suppress and could only be controlled by the more potent TNFR2-positive Tregs. These data are reminiscent of the inherent ability of memory T cells to resist Treg suppression (125), although it was not determined whether TNFR2-positive Tcon cells represent memory T cells (124).

### Intracellular Signaling Molecules Linked to Tcon Resistance

#### Cbl-b

Cbl-b is an E3 ubiquitin ligase that catalyzes the ubiquitylation of target proteins, which can result in their degradation by the proteasome, translocation inside the cell, or alteration in function (126). In T cells, Cbl-b sets the threshold for weak antigen stimulation (127) and enforces the need for costimulation, or “signal 2,” by regulating CD28 signaling (128). Cbl-b negatively regulates the recruitment of the p85 subunit of PI3K to CD28, thereby enforcing T cell anergy and tolerance when signal 2 is lacking (129). Upon CD28 signaling, Cbl-b itself becomes ubiquitylated and degraded, allowing PI3K recruitment and other downstream signaling required for full T cell activation (130). Consistent with its negative regulatory functions, Cbl-b knockout (KO) mice develop systemic autoimmunity due to hyper-proliferation and increased activation of lymphocytes, with T cells that can be activated in the absence of CD28 costimulation (131). Cbl-b KO Tregs were found to be normal, whereas Tcon cells were found to resist suppression by both wild type and Cbl-b KO Tregs, *in vitro* (132) and *in vivo* in a GVHD model (133). In addition to CD4^+^ T cells, Cbl-b KO CD8^+^ T cells also resisted Treg-mediated suppression, providing a mechanism by which Cbl-b KO mice were able to spontaneously reject different types of xenograft tumors as well as ultraviolet-B light-induced skin cancer (134, 135). While the exact downstream mechanism of resistance in Cbl-b KO Tcon cells remains unclear, it is notable that Cbl-b KO T cells showed enhanced PI3K/Akt activation (129).

#### TRAF6

TRAF6 belongs to the E3 ubiquitin ligase family and transduces signals downstream of members of the TNFR superfamily, including IL-1R/TLRs (136), thereby activating NFκB, NFAT, MAP kinases, and Akt signaling pathways (136, 137). A 2006 study demonstrated that TRAF6 KO mice developed multi-organ inflammatory disease characterized by hyper-activated T cells (138). Using mice in which TRAF6 was specifically deleted in T cells, the group showed that while TRAF6 KO Tregs were normal, the Tcon cells resisted Treg suppression both *in vitro* and *in vivo* (138). Re-expression of TRAF6 *via* retroviral transduction restored susceptibility of Tcon cells to Treg-mediated suppression (138). Like Cbl-b KO T cells, TRAF6 KO T cells could also be activated independently of CD28 costimulation, and showed enhanced Akt activation upon TCR signaling. Importantly, sensitivity to Tregs could by restored by overexpression of PTEN, an inhibitor of PI3K/Akt (138). These findings were also supported by human studies indicating that T cells from SLE patients had reduced induction of TRAF6 mRNA upon TCR stimulation, which correlated with increased levels of phospho-Akt and resistance to Treg suppression (116).

#### SHP-1

SHP-1, a protein tyrosine phosphatase, negatively regulates TCR signaling by dephosphorylating signaling mediators such as Zap70, Vav, Lck, and SLP76 (139). Many studies have demonstrated the ability of SHP-1 to regulate the threshold for TCR signaling [reviewed in Ref. (139)] and influence peripheral T cell activation and differentiation (140–143). SHP-1 KO mice develop inflammation in skin and lungs due to myeloid hyper-proliferation (144, 145). These mice also accumulate memory T cells, and T cells are hyper-responsive to TCR stimulation (142, 146–148). We have previously reported that SHP-1 KO Tregs have an increased suppressive capacity (149). Recently, we found that Tcon cells deficient in SHP-1 *via* genetic deletion or pharmacological inhibition can resist Treg suppression *in vitro* (150). SHP-1 has been described as a negative regulator of PI3K/Akt signaling (151), providing a possible mechanism for increased activation and resistance to Treg suppression. SHP-1 also negatively regulates activation of STAT3 in response to IL-6 signaling, with SHP-1-deficient cells being hyper-sensitive to IL-6 (143). Therefore, SHP-1 deficient Tcon cells may be more responsive to IL-6, resulting in activation of STAT3 and subsequent activation of PI3K/Akt. Like Cbl-b KO Tcon cells, SHP-1-deficient CD8^+^ T cells proved an effective method for improving anticancer cytotoxicity (152, 153) (see Employing Tcon Resistance for Cancer Immunotherapy). Whether the enhanced antitumor activity was attributable to Tcon cells resisting Treg suppression remains to be addressed.

Tcon cells from the three aforementioned genetic KO models share the ability to become activated and proliferate with decreased dependence on CD28 costimulation (131, 138, 154). This suggests that the perturbed signaling allows the cells to bypass the need for costimulatory signals that would ultimately activate PI3K/Akt and allow subsequent proliferation. Not only does this provide a means of identifying potentially Treg-resistant Tcon cells as those that do not require costimulation but also reinforces the concept that the PI3K/Akt pathway is hyper-active in Treg-resistant Tcon cells.

### PI3K/Akt: Node of Convergence

Many of the above discussed studies directly demonstrated hyper-activation of the PI3K/Akt pathway in Tcon cells that resist Treg suppression. Evidence is accumulating to suggest that increased PI3K/Akt signaling may be at the heart of Tcon resistance. Wohlfert (155) was the first to propose that the PI3K/Akt pathway was central in allowing Tcon cells to resist suppression. Furthermore, murine models with genetic deficiencies in molecules that negatively regulate the PI3K pathway exhibit Tcon cells resistant to suppression (132, 138, 150). Most compelling is the finding that inhibitors of PI3K and/or Akt can reverse Tcon cell resistance to Treg suppression, making both mouse and human Tcon cells once again susceptible to suppression. This has been accomplished in several ways: by overexpressing the phosphatase PTEN (which antagonizes the activity of PI3K) (138), by using pharmacological PI3K inhibitors wortmannin and Ly294002 (52), by using Akt inhibitors (Akt inhibitor VIII) (24, 31, 116), or by inhibiting cytokine signaling thereby decreasing Akt activation (25). Importantly, carefully titrated inhibition of PI3K and/or Akt did not affect the baseline proliferation of resistant Tcon cells, but instead returned their full susceptibility to suppression by Tregs (24, 25, 52, 138).

It is unknown how increased activation of the PI3K/Akt pathway allows Tcon cells to overcome suppression, especially because the specific mechanisms of suppression employed by Tregs in a given setting vary. In T cells, signaling through the TCR and CD28 rapidly recruits and activates PI3K, but cytokines and other costimulatory receptors can similarly activate PI3K (156). Lipid second messengers produced by activated PI3K bind to Akt and relocate it to the plasma membrane, where it becomes primed for activation (157). Upon activation, Akt promotes proliferation by increasing cell size, inactivating cell cycle inhibitors, and increasing glucose metabolism, as well as enhancing cell survival and allowing cytokine production (158). Mice in which T cells overexpress constitutively active PI3K or Akt develop lymphadenopathy and autoimmunity, underscoring the importance of regulated PI3K/Akt signaling in T cells (158, 159). Inhibition of pro-apoptotic factors such as Bim and the expression of antiapoptotic factors such as Bcl-xL or Bcl-2 are downstream consequences of Akt activation, and a possible mechanism by which Tcon cells escape Treg suppression (55, 68, 116). However, there is little evidence of Tcon cell apoptosis observed under *in vitro* suppression assay conditions, suggesting that alternative suppression mechanisms are overcome by PI3K/Akt activation (52). Both Cbl-b KO and TRAF6 KO Tcon cells, which resist suppression, were still susceptible to Fas-mediated apoptosis (131, 138). Taking these studies into account, although PI3K/Akt activation enhances Tcon cell survival, it does not seem to be the main mechanism by which Tcon cells resist Treg suppression.

Bypassing the need for costimulation is a likely candidate mechanism by which Tcon cells with hyper-activated PI3K/Akt can overcome Treg suppression. Tregs employ various molecules to effectively inhibit APC costimulation of Tcon cells (2). For example, Tregs express CTLA-4, which binds to costimulatory B7 molecules (CD80, CD86) on APCs, leading to their downregulation and preventing Tcon cell costimulation (160). Similarly, LAG3 on Tregs inhibits maturation of DCs to prevent them from activating Tcon cells (161). Thus, engagement of CD28 with CD80 is inhibited, and Tcon cells fail to receive costimulation and subsequent PI3K/Akt activation (13). Treg deprivation of costimulatory signaling would not affect genetically modified Tcon cells that do not require costimulation for full activation, such as Cbl-b, SHP-1, or TRAF6 KO Tcon cells. Furthermore, Treg-resistant Tcon cells from autoimmune diseases may receive adequate stimulation of the PI3K/Akt pathway through other means, such as cytokine, TLR, or TNFR signaling, eliminating the need for costimulation. In this way, any dysregulation of signaling events that lead to hyper-activation of PI3K/Akt can bypass those types of Treg suppression that are mediated by interference of costimulation. While this may not be the only suppressive mechanism overcome by PI3K/Akt hyper-activation, it is certainly a relevant suppressive mechanism both *in vitro* and *in vivo* (13, 161). Akt inactivates FOXO transcription factors, thereby allowing increased cellular metabolism and concomitant entry into cell cycle (158). Thus, another possible mechanism to interrogate is whether enhanced PI3K/Akt signaling results in metabolic changes in Tcon cells that might allow resistance to Treg suppression.

It is important to note that resistance to suppression occurs in both naive and memory Tcon cells (24, 52, 133), and that hyper-activation of PI3K/Akt induces resistance in both subsets (52). Future studies should investigate which suppressive mechanism(s) Tcon cells are able to overcome when PI3K/Akt is hyper-activated, and whether these differ depending on the subset of Tcon cell. Interestingly, murine Tcon cells rendered hyper-responsive by NFATc2/NFATc3 double KO were also able to resist Treg suppression and become activated independently of CD28 costimulation (162). NFAT proteins are regulators of T cell activation, inducing transcription of genes necessary for T cell responses (162). However, the findings of this study suggest that NFATc2/NFATc3 also play a regulatory role in T cell activation, representing a signaling pathway aside from PI3K/Akt that can render Tcon cells resistant to suppression. This finding warrants further investigation into the signaling events that allow Tcon cells to become Treg-resistant, and whether there is a common molecular mediator downstream of both the PI3K/Akt and NFAT pathways.

## Employing Tcon Resistance for Cancer Immunotherapy

Many cancers develop within an immunosuppressive tumor microenvironment, which is detrimental to antitumor immunity. Thus, the ability to induce Tcon cells to resist Treg-mediated suppression would be a desired outcome for immunotherapy. There are several barriers to successful control and/or eradication of tumors, owing to the complex mechanisms that tumors employ to evade the immune system. First, the ability of T cells, namely CD8^+^ CTLs, to recognize antigen on tumors is impaired because tumor cells can decreased expression of MHC I, and because ongoing immune surveillance leads to tumor immunoediting (163). Furthermore, many tumor-associated antigens are in fact self-antigens, to which T cells remain tolerant through peripheral tolerance mechanisms, such as Treg suppression (163). Even when a T cell recognizes a tumor-associated antigen, lack of costimulatory signals prevents effective priming of the T cell. The preponderance of TGF-β secreted by many tumors not only suppresses T cell activation but can also convert T effector cells into Tregs (164). Tregs are enriched in tumors, through chemokine-mediated trafficking to tumors, *de novo* generation, and preferential expansion due to the cytokine environment (164). In many cases, the ratio of Treg/Teff cells is a prognostic indicator, with greater numbers of Tregs indicating a poorer prognosis (164).

Given these obstacles, treatment strategies have attempted to overcome Treg suppression and increase the activation and number of cytotoxic T cells (CTLs) in the tumor. Treg depletion *via* anti-CD25 antibodies or inhibition of Treg function (through antibodies against molecules like CTLA-4) have had some success in boosting antitumor immunity, but typically require combination with tumor vaccines to be highly effective (164, 165). Problematic to these treatments is that Treg depletion is transient and Tregs recover quickly, and some depletion agents can also destroy T effector cells (164). Adoptive cell transfer (ACT) (163) is another current treatment strategy, using patient-isolated tumor-specific CD8^+^ T cells and expanding them *ex vivo* typically with IL-2 or other cytokines. However, ACT is not always effective because transferred T cells do not persist well *in vivo* without the addition of exogenous cytokines, which can have adverse effects (166). Tregs and the immunosuppressive tumor environment also impact the sustained function of the transferred CTLs (167). Thus, investigators have begun to take advantage of the ability to enhance T cell signaling pathways to increase Tcon cell responsiveness (and, potentially, induce resistance to Treg suppression) for use in cancer immunotherapy. To create more potent tumor-specific T cells that can be activated even in a suppressive microenvironment, chimeric antigen receptor (CAR) T cells are being utilized (168). This approach has made use of intracellular signaling domains of costimulatory molecules in order to make the modified T cells hyper-responsive. One strategy was to fuse the intracellular domains of CD28 and the CD3ζ chain to an extracellular, CD19-targeting Ab (to recognize leukemic B cells), resulting in human CAR T cells with enhanced proliferation, resistance to suppression by Treg cells *in vitro*, and acquisition of cytotoxic activity (169). The previous generation of CAR contained only the CD3ζ fused to the CD19-recognizing Ab and also exhibited cytotoxic activity, but could not resist Treg suppression. Although not assessed, it is likely that signaling events downstream of CD28 were enhanced, such as PI3K/Akt, which may have conferred Treg resistance. Therefore, the possibility of inducing T cells to become resistant to Treg suppression and combining this with ACT or other immunotherapies is an attractive solution.

Many of the molecules discussed above that regulate Tcon cell resistance to Treg suppression have also been investigated for their role in antitumor immunity. One way to overcome the need for costimulation is by eliminating Cbl-b. Cbl-b KO mice spontaneously rejected TC-1 tumors and UVB-induced skin tumors (135), as well as thymomas (134), due to increased CD8^+^ T cell tumor infiltration and enhanced cytotoxicity. Importantly, despite there being a greater number of Tregs present in these tumors compared to wild type, the CD8^+^ T cells were resistant to Treg suppression (134, 135). Cbl-b KO CD8^+^ T cells also inhibited the growth of disseminated leukemia (170) and melanoma (171) in mice. These studies clearly demonstrated the advantages to using T cells that have a lower threshold for activation, increased survival, and resistance to Treg- and TGF-β-mediated suppression in order to control tumor growth. It remains to be elucidated how T cell resistance to Treg suppression contributes to tumor control compared to simple hyper-responsiveness of the T cells, and whether or not resistance and hyper-responsiveness are two distinct characteristics of the T cells or represent an overall phenotype.

Similar to Cbl-b KO CD8^+^ T cells, SHP-1 KO CD8^+^ T cells also showed enhanced proliferation without the need for IL-2 supplementation (152). In a mouse model of disseminated leukemia, adoptively transferred SHP-1 KO CD8^+^ T cells decreased tumor size and increased survival rate, with the T cells demonstrating increased cytotoxicity and enhanced survival (152). These results were recapitulated by adoptive transfer of tumor-specific T cells that underwent shRNA knockdown of SHP-1 (152). Similarly, a pharmacological inhibitor of SHP-1, sodium stibogluconate (SSG) showed improved antitumor immunity in mice in a T cell-dependent manner (172), which led to phase I clinical trials of treating advanced cancer patients with a combination therapy of SSG and IFN-α (173, 174). While these studies did not directly assess the influence of Tcon resistance to Treg suppression on tumor control, our studies (150) suggest that SHP-1 KO T cells and Tcon cells from mice treated with SSG do in fact resist Treg suppression and would likely provide an additional advantage for enhanced tumor control.

As discussed above, TLR2 signaling inhibits Treg suppression and also confers Tcon cells with resistance to suppression. Not surprisingly, administration of a TLR2 ligand with an oncoprotein vaccine expanded T effector cells in the presence of Tregs and increased median survival of tumor-bearing mice (81). T effector cells became resistant to Treg suppression, upregulated Bcl-xL, and produced increased cytokines (81). The effect was only elicited by the combination of a TLR2 ligand and the oncoprotein vaccine, but not by either alone. Similarly, in mice immunized with the tumor antigen mERK2 along with plasmids encoding GITR-L, antigen-specific CD8^+^ T cells were capable of inhibiting tumor growth and resisted Treg suppression (108). In a CT26 tumor model, GITR agonist rendered CD4^+^ T cells resistant to suppression and capable of tumor control, as well (175). OX40 signaling prior to tumor challenge also provided tumor control, but in a Treg-dependent manner (101). In this model, OX40 signaling inhibited Treg suppressive function, while also boosting CD8^+^ T cell effector function (101). This provides yet another example of the superior efficacy of treatments that not only inhibit Treg suppressive function but also simultaneously boost T effector function.

PD-1 signaling in T cells is an inhibitory pathway linked to the maintenance of tolerance by blocking T cell activation and downregulating PI3K/Akt signaling (176). While beneficial in preventing autoimmune disease, many tumors cells express high levels of PD-L1 to evade an immune response (177). In addition, there is increasing evidence that Tregs potentiate expression of PD-L1 on APCs as a mechanism to suppress tumor-specific CD8^+^ T cell responses (177–179). In fact, tumor infiltrating CD8^+^ T cells show increased expression of PD-1, which is characteristic of unresponsive, “exhausted” T cells (178, 180). Thus, PD-1 blocking antibodies have recently shown great clinical success in the treatment of metastatic melanoma and non-small cell lung cancer (181), and may also prove successful in other cancer types. Inhibition of this pathway resulted in greater human CD8^+^ T cell differentiation into melanoma-specific CTLs even in the presence of Tregs, conferring them with resistance to PD-1/PD-L1-mediated Treg suppression. Moreover, since PD-1 is critical for Treg function, inhibition of this pathway also interferes with Treg function (178, 179). Therefore, PD-1 blockade antibody therapy has been found particularly useful in combination with other immunotherapeutic modalities (180), as a way to invigorate the effector T cell response in a manner that overcomes Treg suppression while at the same time inhibiting Treg function.

The above studies are consistent with the idea that increased activation of the Akt pathway allows T cells to resist Treg suppression, and that T cells resistant to suppression are better able to control tumor growth. Indeed, human CD8^+^ T cells transduced with constitutively active Akt (caAkt) had enhanced cytotoxicity toward neuroblastoma (182). The caAkt T cells showed increased proliferation and survival, and were resistant to Treg suppression, and had reduced susceptibility to TGF-β-induced conversion into Tregs (182). Future strategies for cancer immunotherapy should take into consideration the importance of inducing T cells to resist suppressive mechanisms and strive to better understand how Treg resistance re-shapes the immune response. Furthermore, current therapies may actually, in part, act by inducing Tcon resistance to Treg suppression, which is worth examining. Suited to the era of personalized medicine, therapies that induce Tcon resistance would be most beneficial in patients whose tumors have a high degree of Treg infiltration or a highly suppressive tumor microenvironment.

## Remaining Questions

While the characterization of the phenomenon of Tcon cells resisting Treg-mediated suppression has come a long way in the past decade, there are still several important questions left unanswered.

### Where Does the Acquisition of Resistance Occur?

In autoimmune diseases, the local inflammatory environment enables Tcon cells to become resistant to suppression. However, there are also examples of Tcon cells acquiring resistance to suppression in the absence of inflammation, when TCR signaling is dysregulated (see Table 1). For example, Tcon cells isolated from the spleen or lymph nodes of mice with a T cell-specific SHP-1 deletion are resistant to Treg suppression *in vitro* (150). Furthermore, CD8^+^ T cells targeted with siRNA to knockdown either Cbl-b or SHP-1 acquire resistance to Treg suppression (152, 170), suggesting that at least under conditions of deficient regulatory molecules, T cells do not require an inflammatory environment to become Treg-resistant. While not necessarily physiological, genetic deficiencies of intracellular signaling molecules have provided information about the mechanism of Tcon resistance and the pathways involved. It is possible that as a result of strong inflammatory signals received by a Tcon cell during autoimmune disease, molecules such as Cbl-b or SHP-1 are sequestered or degraded, so that they no longer regulate T cell signaling. Although this remains to be seen, the fact that Tcon cells can acquire resistance in a TCR-signaling-dependent manner in genetic KO models suggests that acquisition of resistance might occur in secondary lymphoid organs (SLOs).

Studies of autoimmune disease in mice have demonstrated that Tcon cells isolated from sites of inflammation, as well as those from SLOs, are resistant to suppression. Similarly, Tcon cells from peripheral blood of autoimmune disease patients have been found to be resistant to Treg suppression. It is therefore difficult to determine whether Tcon cells became resistant in the inflamed tissue (e.g., synovium, pancreatic islets, CNS) and are re-circulating, or whether they acquired resistance in an SLO upon antigen and/or cytokine encounter. It appears that when certain conditions are met during TCR stimulation, such that the PI3K/Akt pathway becomes hyper-activated, a Tcon cell can become resistant to suppression. Given the number of documented pathways by which a Tcon cell can become resistant to suppression, it would seem that there is opportunity for naive T cells, as well as differentiated effector and memory T cells, to acquire resistance, albeit possibly in different locations. It is likely that naive Tcon cells acquire resistance in SLOs, as they would be primed in the SLO and have yet to traffic to a site of inflammation. Resistant T effector cells that are isolated from active disease settings may represent naive Tcon cells that acquired resistance in an SLO, became activated, and subsequently trafficked to a particular tissue, or may represent cells that became resistant in the inflamed tissue. It will be difficult to determine the location of acquisition of resistance in particular, but use of more sophisticated animal models in conjunction with *in vivo* imaging of Tcon cell activation status should help gain further insights. It is clinically relevant to pinpoint the location of acquisition of resistance in order to employ targeted therapeutic approaches, such as nanoparticle-directed delivery (183) of a compound that could reverse resistance in autoimmunity, or intratumoral injection of a compound to induce resistance in cancer (184).

### How Stable Is the Treg-Resistant Phenotype?

When Tcon cells become resistant to Treg suppression, they undergo cell-intrinsic changes that mediate their resistance. Because of the limitations of *in vitro* suppression assays, many studies have assessed Tcon cell resistance *in vivo*. Tcon cells deficient in TRAF6 or Cbl-b maintain Treg resistance when transferred into a host mouse, as demonstrated by induction of colitis (138) and GVHD (133) in the presence of otherwise protective Tregs. Perhaps not surprisingly, this suggests that despite removal from the inflammatory environment in which they developed, Tcon cells genetically deficient in specific molecules maintain resistance to Treg suppression. Likewise, CD8^+^ T cells lacking Cbl-b or SHP-1 maintain resistance *in vivo* despite their accumulation in a highly suppressive tumor microenvironment, and can successfully control tumor outgrowth (152, 170).

There may be qualitative differences in just how stable the Tcon cell resistance program is, depending upon the circumstances of acquisition. Ideally, for a Tcon cell to respond to a pathogenic threat, it would transiently need to resist Treg suppression. Thus, an abundance of pro-inflammatory cytokines would drive the Tcon cell to resist suppression, perhaps through activation of PI3K/Akt signaling. When the cytokine concentration is reduced as the threat is cleared, signaling would wane and Tcon cells would once again be suppressible. Based on this paradigm, Tcon cells that become resistant in autoimmune disease likely stay that way because of aberrant and chronic cytokine production, the presence of self-antigen, and feed-forward autocrine loops. Tcon cells isolated from JIA patients maintained *in vitro* resistance to Treg suppression, producing high amounts of pro-inflammatory cytokines after 4 days in culture, likely reinforcing their own resistance through PI3K/Akt signaling (24, 25). However, blockade of IL-6 or TNFα signaling, or inhibition of Akt, could restore susceptibility to suppression (24, 25). Interestingly, Tcon cells isolated from MS patients have an accelerated kinetics of IL-6 production and resist Treg suppression and maintained resistance even after being cultured for 24 h in the absence of any cytokines (31). This is consistent with the idea that the cells may continue to produce excess cytokines to maintain a state of resistance, unless their ability to receive those signals is blocked, or PI3K/Akt is inhibited. Indeed, it was recently found that CD8^+^ T cells from the SF of JIA patients were able to self-sustain resistance to suppression by secreting large amounts of IFNγ, and only antibody blockade of IFNγ could restore susceptibility to suppression (185). Overall, the Treg-resistant phenotype of Tcon cells appears to be relatively stable, able to persist in the absence of pro-inflammatory cytokines or other resistance-inducing factors. Future studies will need to assess the ability of Tcon cells to maintain Treg resistance, especially in light of efforts to use adoptive Treg therapy for treatment of autoimmune diseases (186). Infusion of Tregs into patients with Tcon cells resistant to suppression might prove to be ineffective, and should be examined further. Additionally, the stability of induction of Tcon cell resistance to suppression *ex vivo* should be investigated to determine if Tcon cells can maintain resistance in a suppressive tumor microenvironment for cancer immunotherapy.

### What Is the Time Window for a Tcon Cell to Become Resistant?

*In vitro*, there seems to be a limited window of time during which a Tcon cell can resist Treg suppression. Whether a Tcon cell will become successfully activated and be able to proliferate or instead be suppressed by a Treg occurs early on in coculture, within the first 6–12 h (41). Addition of pre-activated murine Tregs to culture with murine Tcon cells after 12 h could not induce suppression of Tcon proliferation, which correlated with the peak of IL-2 production by Tcon cells (41). These findings are consistent with the kinetics of cytokine-induced resistance to suppression observed in Tcon cells from autoimmune disease patients. For example, IL-6 is able to induce human Tcon cells to resist Treg suppression only if given within the first 16 h of coculture. Although there was a modest reduction of suppression if given at 24 h, it was only half as effective as when given at 4 or 16 h of culture (31). Likewise, incubation of human Tcon cells with IL-15 *in vitro* rendered them refractory to suppression owing to increased PI3K/Akt activation (52). In this setting, PI3K inhibitors had to be added to culture within the first 24 h or resistance could not be reversed (52). *In vitro* studies of Treg suppression have provided valuable information regarding the window in which a Tcon cell can become resistant, but the acquisition of resistance *in vivo* is likely a much more complex process. The mechanisms employed by Tregs to suppress Tcon cells *in vivo* are most likely different than *in vitro*, and depend on the anatomical location of the Treg (187). *In vitro*, if a quorum of Tcon cells resist suppression and quickly produce cytokines, this might trigger nearby Tcon cells to also resist suppression as they are concentrated (in a well of a tissue culture dish). This is in contrast to a physiological setting, where only a small subset of T cells might be in close enough proximity to spread resistance *via* cytokine secretion. In the context of autoimmune disease, this begs the question, at what stage do Tcon cells become resistant to Treg suppression, and is it a causative factor of the disease or a consequence? If Tcon cells in autoimmune disease settings become resistant due to a preponderance of inflammatory cytokines, this would suggest that the disease must already be underway before resistance is induced. Indeed, Tcon cells from patients with inactive lupus nephritis showed a higher level of activated Akt compared to healthy control cells, but not as high as that from patients with active lupus, suggesting that the degree of resistance corresponds to severity of disease (116). Therefore, a break in tolerance may be responsible for autoimmune disease initiation, but as the disease progresses, Tcon cells become Treg-resistant, exacerbating disease severity. It is yet to be determined whether *in vivo* treatment with PI3K and/or Akt inhibitors could reverse Treg resistance in established autoimmune disease, or whether there is only a short window during disease progression in which Tcon cell resistance can be blocked. This is not easily answered, as therapeutic PI3K/Akt inhibitors are currently unavailable. However, successful treatment of MS and RA/JIA symptoms using anti-IL-6 or anti-TNF therapy suggests that the cycle of Tcon cell resistance *in vivo* can be broken during ongoing disease (60, 63), and T cell-specific manipulation of PI3K/Akt pathway might be a future option for the treatment of autoimmune diseases and/or tumor immunotherapy.

## Concluding Remarks

Deepening our understanding of what determines the susceptibility of a Tcon cell to Treg-mediated suppression will prove extremely useful in advancing therapies for both autoimmunity and cancer. Although there are various mechanisms employed by Tregs to suppress Tcon cells, the PI3K/Akt pathway is a downstream point of convergence, representing an ideal therapeutic target. Already, efforts have been made to utilize Tcon cells resistant to suppression in controlling tumor outgrowth, and have shown promise as part of a combinatorial therapy. Further improvements upon autoimmune disease treatments could be made if the PI3K/Akt pathway could be specifically inhibited in out-of-control Tcon cells in order to rein them in. Finding the appropriate balance between Tregs and Tcon cells in different settings remains elusive, but further studies addressing the questions posed in this review will allow better manipulation of the delicate balance between Tregs and Tcon cells.

## Author Contributions

Both authors contributed to the inception, writing, and editing of the review.

## Conflict of Interest Statement

The authors declare that the research was conducted in the absence of any commercial or financial relationships that could be construed as a potential conflict of interest.

## Abbreviations

γ_c_, common gamma chain; APC, antigen presenting cell; CFSE, 5,6-carboxyfluorescein diacetate succinimidyl ester; CNS, central nervous system; CTL, cytotoxic T lymphocyte; DC, dendritic cell; EAE, experimental autoimmune encephalitis; JIA, juvenile idiopathic arthritis; KO, knockout; MS, multiple sclerosis; PI3K, phosphatidylinositol 3-kinase; RA, rheumatoid arthritis; SF, synovial fluid; SLE, systemic lupus erythematosus; T1D, type 1 diabetes; Tcon, conventional T cell; TCR, T cell receptor; TLRs, toll-like receptors; TNFRs, tumor necrosis factor receptors; Treg, regulatory T cell.
